# Supplementary material for: CryoEM Structures of Antibodies Elicited by Germline-Targeting HIV MPER Epitope-Scaffolds
Source: bioRxiv. 2025 Aug 19:2025.08.19.671101. Preprint. [Version 1] doi: 10.1101/2025.08.19.671101 (PMC12393349; doi:10.1101/2025.08.19.671101)
Supplement: Supplement 1 [file media-1.pdf]

Supplemental Information

CryoEM Structures of Antibodies Elicited by Germline-Targeting HIV MPER Epitope-Scaffolds

Jiachen Huang[1], Olivia M. Swanson[1,2,3,4], Kimmo Rantalainen[2,3,4], Monica L. Fernández-Quintero[1],
Johannes R. Loeffler[1], Ryan Tingle[2,3,4], Erik Georgeson[2,3,4], Nicole Phelps[2,3,4],
Gabriel Ozorowski[1,3,4], Torben Schiffner[2,3,4], William R. Schief [2,3,4,5*], Andrew B. Ward[1,3,4*,†]

[1]Department of Integrative Structural and Computational Biology, The Scripps Research Institute, La Jolla, CA, USA
[2]Department of Immunology and Microbiology, The Scripps Research Institute, La Jolla, CA, USA
[3]Consortia for HIV/AIDS Vaccine Immunology and Immunogen Discovery (CHAVD), The Scripps Research Institute, La Jolla, CA, USA
[4]IAVI Neutralizing Antibody Center, The Scripps Research Institute, La Jolla, CA, USA
[5]Moderna, Inc., Cambridge, MA, USA

*Correspondence: schief@scripps.edu (WRS), andrew@scripps.edu (ABW)
†Lead contact: andrew@scripps.edu

Table S1. CryoEM data collection, processing and model building statistics

Model/Map	GT10.2+W3-05+W6-03	GT10.2+W3-02+W6-03	GT10.2+W10-09+W6-10	GT12+10E8+W6-10	GT12+SA2911 + W6-10
**EMDB**	EMD-71883	EMD-71885	EMD-71884	EMD-71882	EMD-71881
**PDB**	9PV5	-	-	9PV4	9PV3
**Data collection & processing**					
**Microscope/ Detector**	Glacios/Falcon 4	Glacios/Falcon 4	Glacios/Falcon 4	Glacios/Falcon 4	Krios/Gatan K3
**Voltage (kV)**	200	200	200	200	300
**Magnification**	190kx	190kx	190kx	190kx	105kx
**Recording mode**	Counting	Counting	Counting	Counting	Counting
**Pixel size (Å)**	0.725	0.725	0.725	0.725	0.833
**Total dose (e^-/Å^2)**	50.4	50.4	46.5	43.2	52.6
**Defocus range (μm)**	-0.7 to -1.5		-0.8 to -1.8	-0.8 to -1.8	-1.0 to -2.0
**No. of movie micrographs**	6090		6918	3585	4104
**No. of molecular projection images in map**	41,951	63,258	12,994	72,061	171,994
**Symmetry**	C1	C1	C1	C1	C1
**Map pixel size (Å)**	0.725	0.725	0.725	0.725	0.833
**Map resolution (FSC 0.143; Å)**	3.93	4.21	6.31	4.01	3.95
**Map sharpening B-factor (Å2)**	-101.8	-162.3	-490.3	-128.2	-170.3
**Structure building and validation**					
**Model composition**					
**Non-hydrogen atoms**	4963	-	-	4831	4862
**Protein residues**	630	-	-	616	618
**Ligands**	NAG:1	-	-	NAG:1	NAG:1
**RMSD bond length (Å)/angles (°)**	0.008/1.459	-	-	0.018/1.749	0.008/1.411
**MolProbity score**	1.63	-	-	1.24	1.72
**EMRinger score**	1.05	-	-	2.89	2.1
**Clash score**	4.92	-	-	3.05	5.64
**Ramachandran outliers/allowed/favored (%)**	0/5.48/94.52	-	-	0/2.81/97.19	0/6.25/93.75
**Rotamer outliers (%)**	0	-	-	0	0.19
**Cβ outliers (%)**	0	-	-	0	0

Table S2. Off-target epitope mapping with patch mutations of GT10.2

	% of GT10v55 binding						
	Patch 1	Patch 2	Patch 3	Patch 6	Patch 7	Patch 8	Patch 9
SA684_Exp1_Scaf_MPER-GT10v55_W6-01	1.13	0.26	1.10	0.96	1.09	0.87	1.09
SA684_Exp1_Scaf_MPER-GT10v55_W6-02	0.93	0.94	0.73	0.77	0.94	0.60	0.62
SA684_Exp1_Scaf_MPER-GT10v55_W6-03	0.90	0.88	0.89	0.90	0.92	0.33	0.28
SA684_Exp1_Scaf_MPER-GT10v55_W6-05	1.29	0.52	1.18	0.76	1.32	0.98	1.16
SA684_Exp1_Scaf_MPER-GT10v55_W6-06	0.88	0.96	0.78	1.00	0.80	0.24	0.26
SA684_Exp1_Scaf_MPER-GT10v55_W6-07	0.83	0.95	0.79	1.02	0.86	0.58	0.87
SA684_Exp1_Scaf_MPER-GT10v55_W6-08	0.84	0.88	0.94	0.93	0.96	0.94	0.90
SA684_Exp1_Scaf_MPER-GT10v55_W6-09	1.22	0.74	1.29	0.94	1.12	0.83	1.15
SA684_Exp1_Scaf_MPER-GT10v55_W6-10	0.96	0.88	0.99	0.94	1.02	0.75	0.73
SA684_Exp1_Scaf_MPER-GT12v56_W6-01	0.68	0.78	0.76	0.80	0.79	0.66	0.48
SA684_Exp1_Scaf_MPER-GT12v56_W6-02	0.93	0.72	0.98	0.81	1.03	0.95	0.92
SA684_Exp1_Scaf_MPER-GT12v56_W6-03	0.85	0.97	0.80	0.99	0.73	0.81	0.77
SA684_Exp1_Scaf_MPER-GT12v56_W6-04	0.90	0.39	0.84	0.96	0.93	0.99	0.90
SA684_Exp1_Scaf_MPER-GT12v56_W6-05	0.86	0.94	0.92	0.96	0.95	0.69	0.95
SA684_Exp1_Scaf_MPER-GT12v56_W6-06	1.29	0.71	1.38	0.89	1.19	0.77	1.24
SA684_Exp1_Scaf_MPER-GT12v56_W6-07	0.97	0.88	0.94	0.95	0.99	0.19	0.25
SA684_Exp1_Scaf_MPER-GT12v56_W6-08	0.96	0.80	0.43	0.87	1.05	0.78	0.91
SA684_Exp1_Scaf_MPER-GT12v56_W6-09	0.96	0.16	0.92	0.90	0.98	0.64	0.36
SA684_Exp1_Scaf_MPER-GT12v56_W6-10	0.90	0.95	0.81	0.98	0.88	0.27	0.46
10E8UCA	0.87	0.84	0.53	0.99	0.90	1.00	0.87

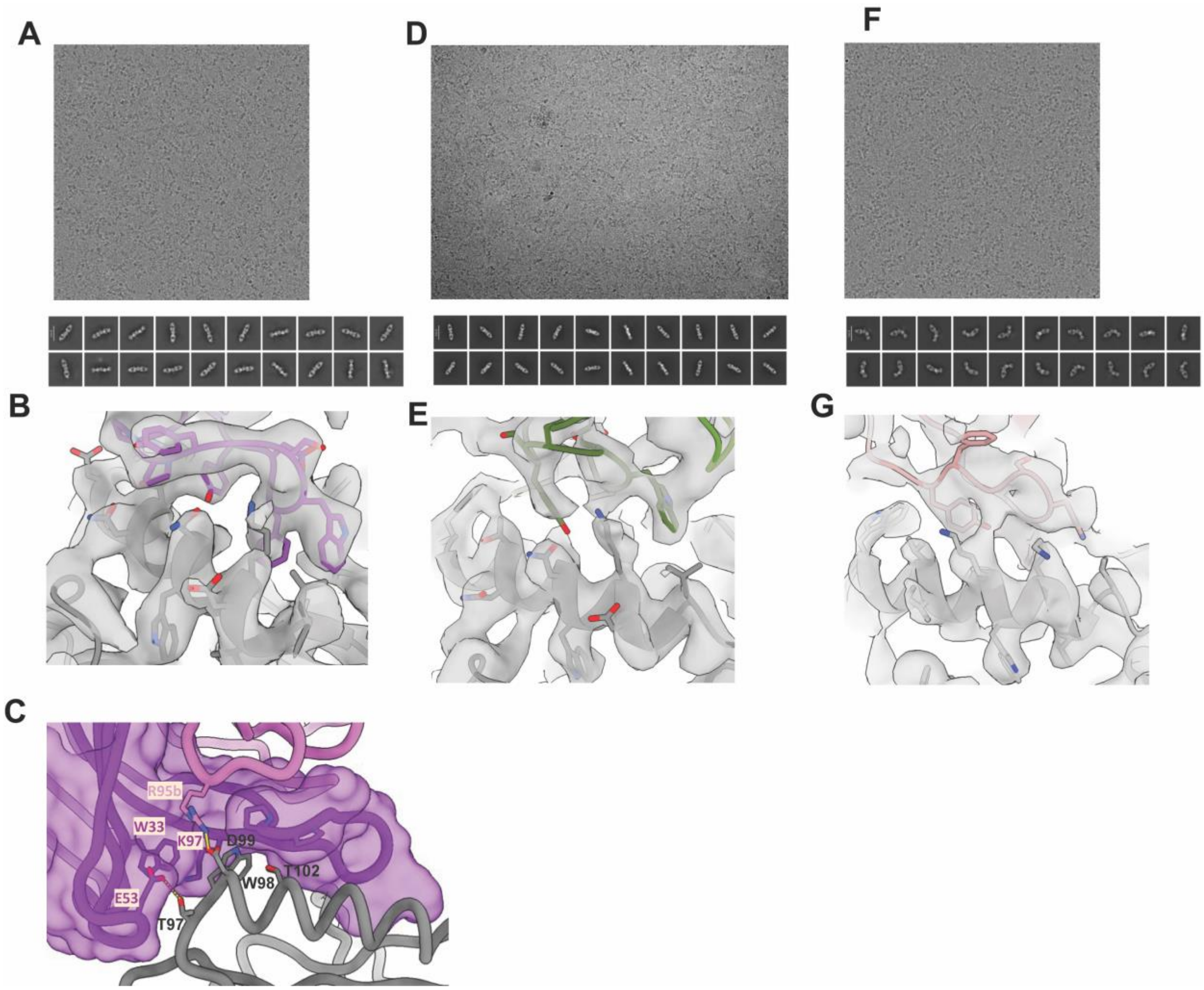

Figure S1. Representative micrograph, selected 2D classes, and density map near MPER helix are shown for 10E8+GT12+W6-10 complex (A & B), SA2911+GT12+W6-10 complex (D & E), and W3-05+GT10.2+W6-03 complex (F & G). (C). GT12 interactions with 10E8 (non-HCDR3 mediated) with 10E8 HC shown with surface.

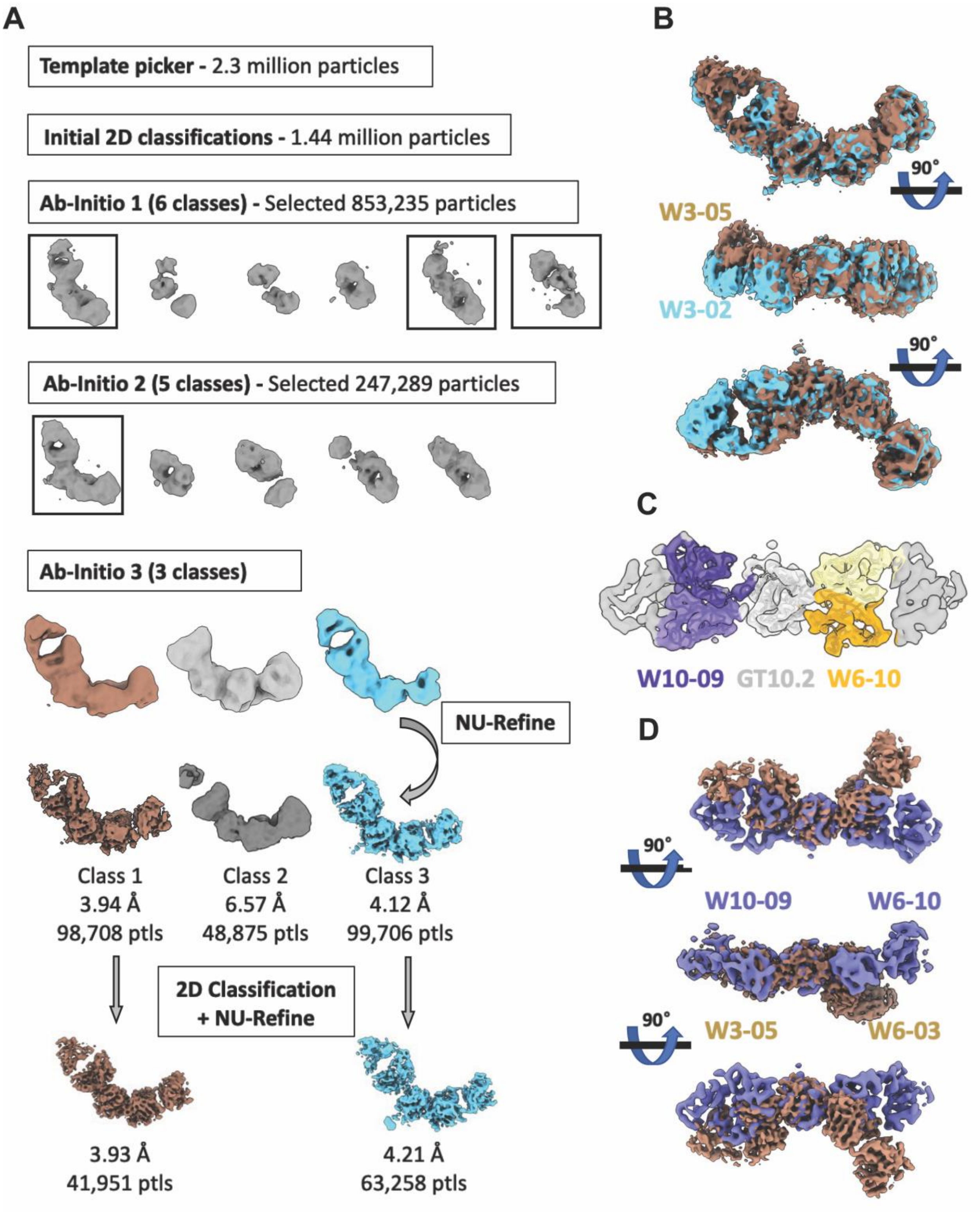

Figure S2. CryoSPARC data processing workflow and map comparisons of the 3 on-target Fab pool dataset. (A). Particles were cleaned up and classified through 2D classification and multiple rounds of Ab-initio jobs and final maps were obtained by non-uniform refinement jobs. (B). Aligned maps from 2 different classes showing the shift of binding angle of the on-target

Fabs. (C). CryoEM map of W10-09 (purple)/W6-10 (yellow) Fabs complexing with GT10.2. (D). Aligned maps of W3-05 + GT10.2 + W6-03 vs. W10-09 + GT10.2 + W6-10 showing different binding angles of on/off-target Fabs.

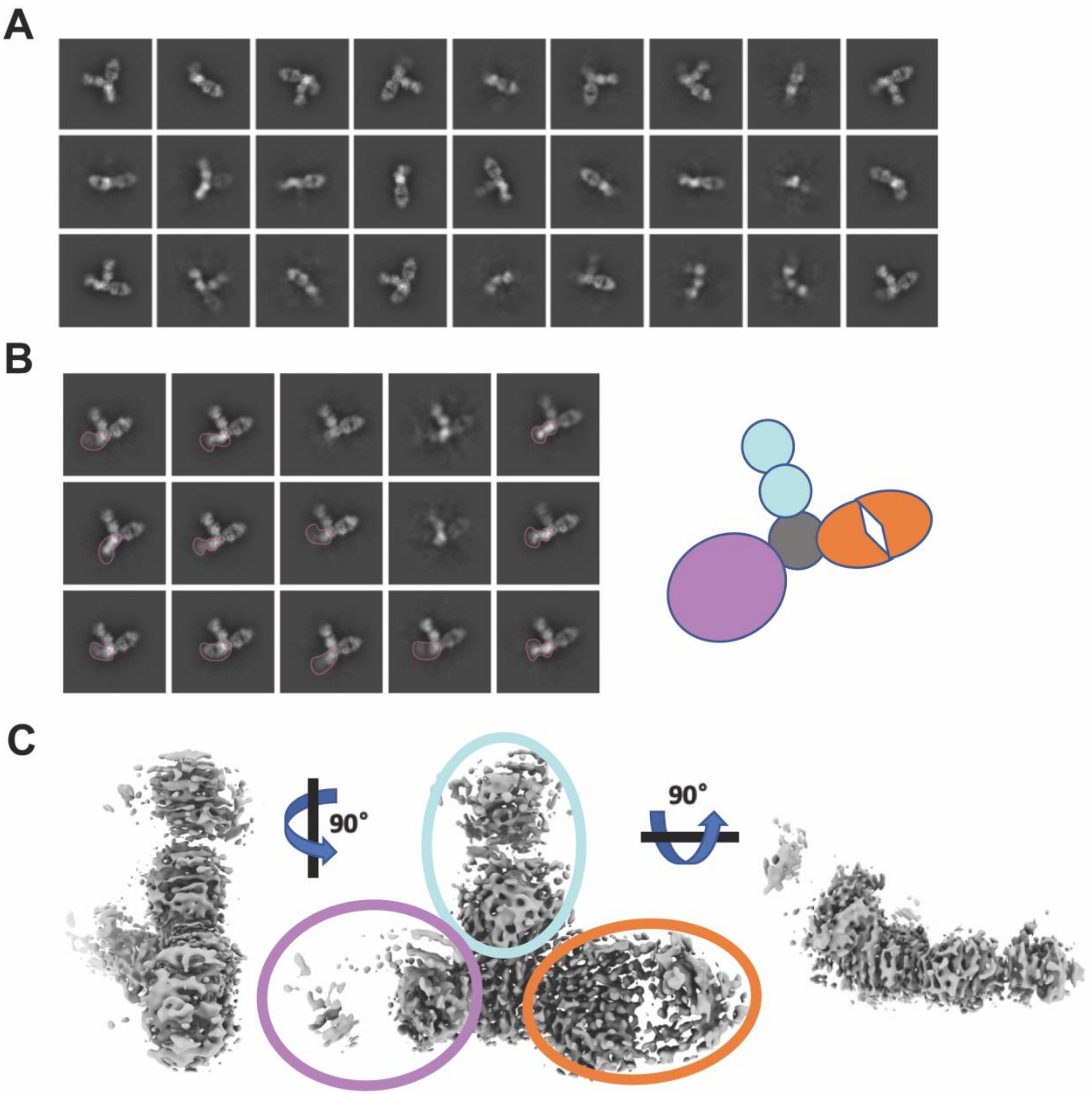

Figure S3. Selected 2D classes and map from 6 on-target Fab pool dataset. Selected 2D classes (A) and representative superclass from a CryoSPARC rebalance 2D classes job (B) showing a variety of binding poses on on-target Fabs (circled in purple) from the pool. (C). A 3D reconstruction map showing Fab densities on 3 different binding sites of HGT10.2 immunogen.

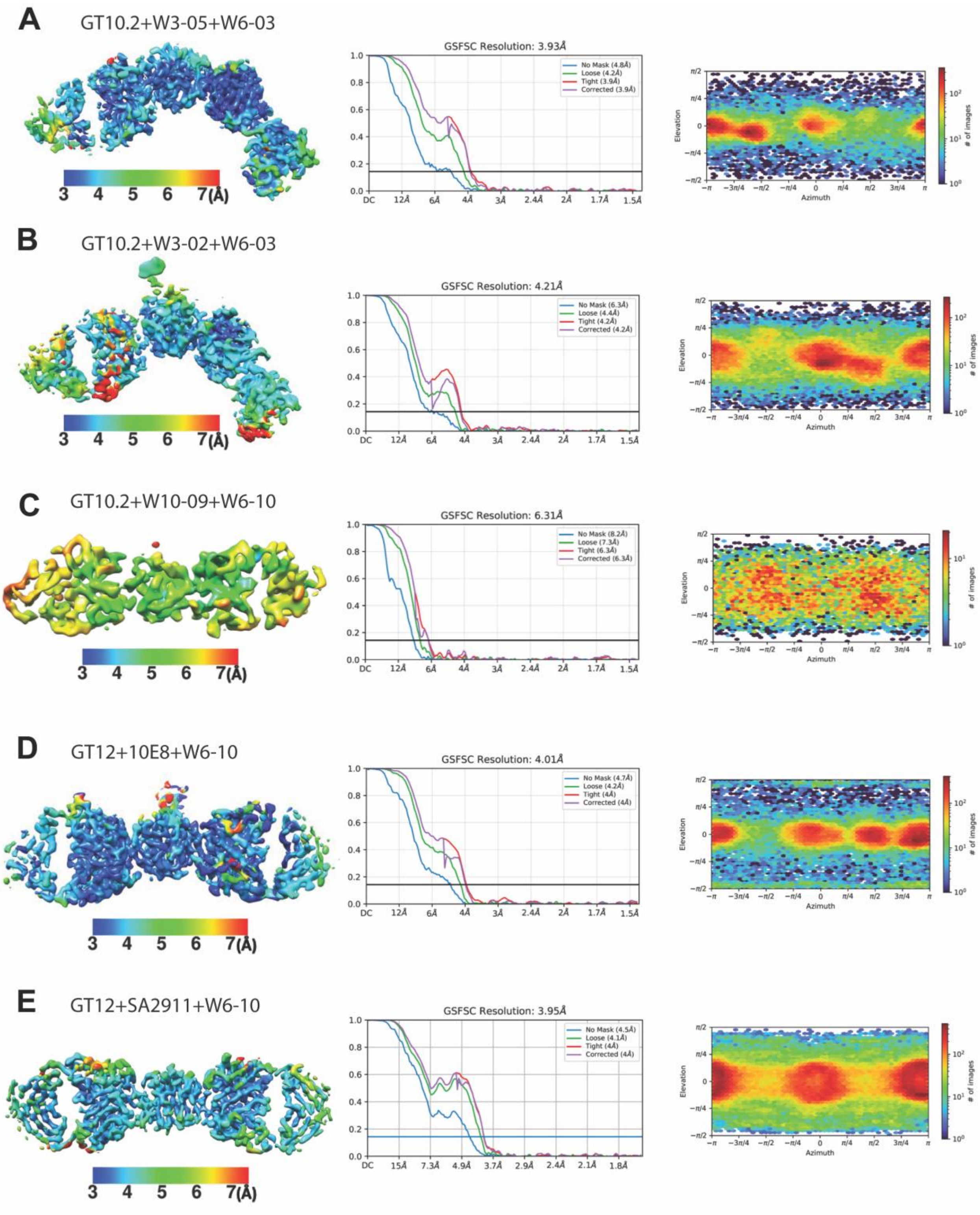

Figure S4. The local resolution maps, Fourier shell correlation curves and angular sampling for cryoEM maps in this manuscript.

Video 1. MD simulation of 10E8+GT10.2

Video 2. MD simulation of 10E8+GT12

Video 3. MD simulation of W3-05+GT10.2

Video 4. MD simulation of SA2911+GT12
